# Comparison between infants receiving traditional supplements (camel thorn, flix weed, and sugar water) and exclusively breast fed infants

**Published:** 2015

**Authors:** Hasan Boskabadi, Sepideh Bagheri

**Affiliations:** Neonatal Research center, Ghaem hospital, School of Medicine, Mashhad University of Medical Sciences***, ****Mashhad, Iran*

**Keywords:** *Breast feeding*, *Neonate*, *Traditional medicine*

## Abstract

**Objective:**

Although breast milk is considered the best nutritional option for neonates, use of traditional supplements such as sugar water, camel thorn, and flix weed in the first week of life of infants is quite common in Iran and many other countries. The aim of this study was to evaluate whether consuming such supplements has any impact on infant’s breastfeeding behavior.

**Materials and Methods:**

Four hundred fifty four term infants who were referred to the neonatal clinic of Ghaem hospital were enrolled and divided into two groups. Control (exclusively breastfed infants, N=243) and case (breast milk feeding plus traditional remedies such as sugar water, camel thorn, and flix weed, N=211). Spss 19.5 was used for statistical analysis. T-test and Man-Whitney tests were used. A p-value of <0.05 was considered as significant.

**Results:**

The two groups were similar in their baseline data. Regarding duration of breastfeeding and breastfeeding frequency, use of these supplements resulted in a reduction in both breastfeeding frequency and duration (p<0.05). Breastfeeding problems such as poor let-down reflex and incorrect breastfeeding position were more common among mothers feeding these supplements to their infants. Moreover, infants with delayed initiation of first breastfeeding were more likely to receive these supplements.

**Conclusion:**

Based on the results of this study, feeding infants with sugar water, camel's thorn, and flix weed is clearly associated with breast feeding problems such as poor let down reflex and incorrect breast feeding position. Use of these supplements resulted in a reduction in frequency and duration of breast feeding. Infants with delayed initiation of breast feeding are more likely to receive these *supplementations*. Therefore, any attempts to improve the community's culture would be of great benefit to the health and well being of our babies.

## Introduction

Breast milk is considered the best nutritional option for infants and provides health advantages to both infant and mother while meeting their emotional needs. It plays a great role in preventing different infections in children especially gastrointestinal and respiratory tract infections (Horta et al., 2007 ▶; IP et al., 2007 ▶; Gartner et al., 2005 ▶). World Health Organization (WHO) emphasizes on breast milk as the best source of nutrition for the ideal growth and development of infants and suggests exclusive breast feeding for the first 6 months of life (Kramer, 2004 ▶). However, in the first few days of life, many factors may interfere with lactation and breast feeding such as breast problems like engorgement and nipple cracks which can lead to inadequate milk intake (Boskabadi et al., 2010 ▶). 

Moreover, mothers may feel uncomfortable while nursing and there is also a traditional belief that feeding the neonate with sugar water, camel thorn (botanical name: Alhaghi pseudoalhaghi), and flix weed (botanical name: descurainia Sophia) can be effective in the prevention and treatment of neonatal jaundice. Because of all these reasons, use of these remedies is quite common in our culture (Boskabadi et al., 2010 ▶). 

Many studies have evaluated the effects of these remedies on neonatal jaundice but adequate attention has not been paid to their nutritional effects. Therefore, we conducted this case-control study to compare exclusively breast fed neonates with those receiving such supplements.

## Materials and Methods

This study was conducted from September 2008 to September 2012. Four hundred fifty four term neonates who admitted the neonatal clinic of Ghaem hospital, Mashhad, north east of Iran, were included in the study. The study was approved by the ethical committee of Mashhad University of Medical Sciences (MUMS) prior to performance and parental informed consent was obtained for every patient before enrollment.

Neonates were excluded if they had any of the following conditions: preterm birth, formula feeding, multiple anomaly, chromosomal abnormalities, neonates with conditions such as meningitis or septicemia, congenital heart disease, and 5-minute Apgar score of less than 7. 

The neonates were divided into two groups according to feeding type: Exclusively breastfed (n=243) as control group vs. those who received supplements (sugar water, camel's thorn, or flix weed) besides breast milk (n=211) as case group.

Neonatal factors (age, birth weight, weight on admission measured by the physician using the same scale for all neonates, 5-minute APGAR score, sex, type of feeding, number of feedings, feeding duration, number of supplementation, and amount of supplement used per day) as well as maternal factors (age, weight, education level, parity, pregnancy complications, mode of delivery, breast problems, breast feeding technique, let-down reflex, time of breast feeding initiation, and frequency of breast feeding) were recorded for all cases and controls.

A breast problem was defined as the presence of one of the followings: inverted nipple, cracked nipple, mastitis, or breast engorgement identified during the physical examination. 

The proper breastfeeding position was identified using WHO breastfeeding observation form.

The let-down reflex was defined as the milk ejection in response to suckling. Let-down reflex was considered positive if the mother could feel the sensation of milk “coming in”.

All these data were collected using a questionnaire filled in by a midwife and all neonates were examined by a neonatologist.

**Statistical Analysis**

All statistical analysis was performed using SPSS 19.5 statistical package. Values were expressed as mean±SD. The group comparisons were assessed by student's t-test (in case of normally distributed data) or Mann-Whitney U test (in case of non- normally distributed data). Categorical variables were compared using chi-square test. A two tailed p-value <0.05 was considered as statistically significant.

## Results

**Maternal findings**

A total of 454 mothers and their neonates participated in this study (either as a member of the control or the case group). No significant difference was observed in age, education, parity, weight, and presence of breast problems between mothers in the control and case group (p>0.05, Table 1). However, the rate of inappropriate breast feeding position was significantly higher in case group (p= 0.000). From 211 mothers of case group, 68 (33.2%) had inappropriate breast feeding position.

**Neonatal findings**

There were no statistically significant differences between the two groups regarding gender, birth weight, weight on admission, and 5-minute Apgar score. 

The average time to the first feed post partum was 1.5 hours in control and 3.4 hours in case group which is significantly different (p= 0.000). The average number of feeds per day was 12.1±5.7 for control vs. 10.2±4.7 for case group which is statistically different (p= 0.004). 

This shows that breastfeeding frequency was reduced by using these supplementations. Moreover, duration of each feeding was shorter in infants who received these supplements (30.53±7.1 min vs. 22.71±4.1 min, p=0.026).

**Table 1:** Breast feeding characteristics of case and control group

	**control**	**Case**	**p-value**
**Inappropriate breast feeding position**	N= 23	N= 68	0.000
**Absent let-down reflex**	N= 53	N= 66	0.009
**Delay in breast feeding initiation **	N= 8	N= 21	0.047
**Presence of breast problems**	N= 10	N= 18	0.823

**Table 2:** Characteristics of case and control infants

	**Control**	**Case**	**p-value**
**Age (day)**	7.4±5.21	7.92±4.33	0.199
**Birth weight (gr)**	3122±536	3152±500	0.552
**Weight on admission (gr)**	3053±593	3091±202	0.808
**Time of first breast feeding (minute)**	90±85	195±186	0.000
**Number of feedings/day**	12.34±3.40	10.28±4.71	0.004
**Duration of feeding (minutes)**	30.53±7.1	22.71±4.1	0.026

## Discussion

Breast feeding is considered the best nutritional source for neonates. The present study reveals that unlike the popular beliefs, traditional supplements such as sugar water, camel's thorn, and flix weed do not improve neonatal weight gaining but also are associated with fewer and shorter breastfeeding episodes. General beliefs are part of each society's culture and are transmitted from elders to youth therefore they are respected by most people (Raven et al. 2007 ▶). These ideas are sometimes useful and sometimes harmful to the health. For example, keeping the neonates warm in the first hours of life by local midwives may help them to accommodate with the new environment (Goldsmith, 2011 ▶). 

However, exaggerating this practice in the form of swaddling the baby increases the risk of hyperthermia and weight loss (Boskabadi et al., 2010 ▶). Knowledge about these traditional beliefs and attempts at improving the wrong ideas through suitable methods would be of great help to the overall health and well being of the society (Boskabadi et al., 2011 ▶). General belief that" feeding infants substances that cause soft stool, will improve jaundice" has led to the use of traditional remedies such as sugar water, camel thorn, and flix weed for infants in our country. In our study, this cultural belief was not influenced by maternal age, education, and parity indicating that this is a serious belief in our community.

Many researchers have evaluated the effects of such substances on neonatal hyperbilirubinemia. In a study by Mathew and Warthon (1981) ▶ about the effect of feeding infants with water on serum bilirubin levels, a significant difference was not observed between two groups. Tarhani and his coworkers (2005) ▶ in Lorestan University studied the effect of camel thorn on neonatal hyperbilirubinemia and showed that this practice does not lower serum bilirubin level significantly. 

Use of sugar water is also common in the first days of life. In this study, 32% of neonates in case group received sugar water. Ansell et al. (1997) ▶ reported that giving sugar water was not successful at preventing or treating dehydration in the neonates. Dewey et al. (2003) ▶ reported that feeding infants non breast milk fluids is associated with lactation failure. 

In this study, newborns received traditional remedies as follows: camel's thorn (63%), sugar water (32.3%), and flix weed (4.7%) and they received these supplements between 3-10 times per day. It seems that special attention needs to be paid to camel thorn and its effects and complications.

In the present study, presence of inappropriate breast feeding position was significantly greater in infants receiving supplements (44% vs. 11% in control group). In a study on neonates with hypernatremic dehydration, this was reported as high as 50% (boskabadi et al., 2010 ▶; Livingstone et al., 2000). Whether it is the inappropriate breast feeding position that leads to the use of traditional supplements or use of these supplements leads to inappropriate breast feeding position is not fully understood but it seems more likely that both conditions aggravate each other. Inappropriate breast feeding position leads to absence of let-down reflex and the infant’s resultant hunger and irritability leads to the use of these supplements. Absence of let-down reflex in our case group can be a confirmation to this theory. On the other hand, feeding these supplements to the neonate using bottles leads to nipple confusion and the infant may bite the breast which leads to sore and cracked nipples and thus aggravates the inappropriate latch (Kramer et al., 2001 ▶). Therefore, it seems that education on the most appropriate position for breast feeding to all new mothers is essential for successful lactation. Mothers must be notified about the risks of using bottles and supplements.

Mode of delivery, pregnancy complications, infant’s gender, and 5-minute Apgar score was not significantly different between two groups which show that neonatal and maternal problems are not important for the decision to use these supplements and cultural beliefs play the most important role. Birth weight and weight on admission was not also different between the two groups and this questions the general idea that inadequate breast milk in the first days of life leads to the use of these supplements.

In the present study, the incidence of delayed initiation of breast feeding was greater in neonates receiving supplements. Delay in the initiation of breast feeding soon after birth can cause breast engorgement thus augmenting lactation problems leading parents to use supplements. In a study, use of supplements in mothers with breast problems was greater. 

Moreover, traditional remedies such as camel thorn, flix weed, and sugar water were used in 85% of such neonates which itself shows the great belief of the society about the effect of these substances (Boskabadi et al., 2014 ▶). Multiple studies could not show any positive effect of such therapies for neonates (Kazerani et al., 2007 ▶; Nabavizadeh et al., 2006 ▶). Moreover, these supplements were not effective in controlling jaundice or infant’s weight loss (Boskabadi et al., 2011 ▶; Panjvani et al., 1995 ▶) and in a study they also led to delayed referral and exaggeration of neonatal jaundice (Boskabadi et al., 2011 ▶).

Frequency of breast feeding was clearly lower in the case group. It may be due to the sweet taste of the supplements which decreases the neonate’s appetite and thus decreases breast milk intake which is higher in calories and may lead to infant’s dehydration too.

Therefore, results of this study show the adverse effects of such therapies on infants breastfeeding behavior which may lead to premature weaning of breastfeeding.

The results of the present study indicate that use of camel thorn, flix weed, and sugar water is associated with decreases in the frequency and duration of breast feeding. Use of supplements was greater among neonates with delayed initiation of breast feeding after birth. Implementation of hospital policies to initiate breastfeeding as soon as possible after birth seems necessary. It is rational to use all society's potentials to modify these cultural beliefs.
